# Clinical Characteristics and Morbidity Associated With Coronavirus Disease 2019 in a Series of Patients in Metropolitan Detroit

**DOI:** 10.1001/jamanetworkopen.2020.12270

**Published:** 2020-06-16

**Authors:** Geehan Suleyman, Raef A. Fadel, Kelly M. Malette, Charles Hammond, Hafsa Abdulla, Abigail Entz, Zachary Demertzis, Zachary Hanna, Andrew Failla, Carina Dagher, Zohra Chaudhry, Amit Vahia, Odaliz Abreu Lanfranco, Mayur Ramesh, Marcus J. Zervos, George Alangaden, Joseph Miller, Indira Brar

**Affiliations:** 1 Department of Infectious Diseases, Henry Ford Hospital, Detroit, Michigan; 2 Department of Internal Medicine, Henry Ford Hospital, Detroit, Michigan; 3 School of Medicine, Wayne State University, Detroit, Michigan; 4 Department of Emergency Medicine, Henry Ford Hospital, Detroit, Michigan

## Abstract

**Importance:**

In late December 2019, an outbreak caused by a novel severe acute respiratory syndrome coronavirus 2 emerged in Wuhan, China. Data on the clinical characteristics and outcomes of infected patients in urban communities in the US are limited.

**Objectives:**

To describe the clinical characteristics and outcomes of patients with coronavirus disease 2019 (COVID-19) and to perform a comparative analysis of hospitalized and ambulatory patient populations.

**Design, Setting, and Participants:**

This study is a case series of 463 consecutive patients with COVID-19 evaluated at Henry Ford Health System in metropolitan Detroit, Michigan, from March 9 to March 27, 2020. Data analysis was performed from March to April 2020.

**Exposure:**

Laboratory-confirmed severe acute respiratory syndrome coronavirus 2 infection.

**Main Outcomes and Measures:**

Demographic data, underlying comorbidities, clinical presentation, complications, treatment, and outcomes were collected.

**Results:**

Of 463 patients with COVID-19 (mean [SD] age, 57.5 [16.8] years), 259 (55.9%) were female, and 334 (72.1%) were African American. Most patients (435 [94.0%]) had at least 1 comorbidity, including hypertension (295 patients [63.7%]), chronic kidney disease (182 patients [39.3%]), and diabetes (178 patients [38.4%]). Common symptoms at presentation were cough (347 patients [74.9%]), fever (315 patients [68.0%]), and dyspnea (282 patients [60.9%]). Three hundred fifty-five patients (76.7%) were hospitalized; 141 (39.7%) required intensive care unit management and 114 (80.8%) of those patients required invasive mechanical ventilation. Male sex (odds ratio [OR], 2.0; 95% CI, 1.3-3.2; *P* = .001), severe obesity (OR, 2.0; 95% CI, 1.4-3.6; *P* = .02), and chronic kidney disease (OR, 2.0; 95% CI, 1.3-3.3; *P* = .006) were independently associated with intensive care unit admission. Patients admitted to the intensive care unit had longer length of stay and higher incidence of respiratory failure and acute respiratory distress syndrome requiring invasive mechanical ventilation, acute kidney injury requiring dialysis, shock, and mortality (57 patients [40.4%] vs 15 patients [7.0%]) compared with patients in the general practice unit. Twenty-nine (11.2%) of those discharged from the hospital were readmitted and, overall, 20.0% died within 30 days. Male sex (OR, 1.8; 95% CI, 1.1-3.1; *P* = .03) and age older than 60 years (OR, 5.3; 95% CI, 2.9-9.7; *P* < .001) were significantly associated with mortality, whereas African American race was not (OR, 0.98; 95% CI, 0.54-1.8; *P* = .86).

**Conclusions and Relevance:**

In this review of urban metropolitan patients with COVID-19, most were African American with a high prevalence of comorbid conditions and high rates of hospitalization, intensive care unit admission, complications, and mortality due to COVID-19.

## Key Points

**Question:**

What are the clinical characteristics and outcomes of patients with coronavirus disease 2019 evaluated at Henry Ford Health System in Southeast Michigan during the early phase of the outbreak?

**Findings:**

In this case series involving 463 consecutive patients with confirmed coronavirus disease 2019 evaluated at a 5-hospital system serving metropolitan Detroit, 72.1% of patients were African American and 94.0% had at least 1 comorbidity. Among the 355 patients who were hospitalized, 39.7% required intensive care unit admission, of whom 80.8% underwent invasive mechanical ventilation and 40.4% died within 30 days.

**Meaning:**

In this series of patients with coronavirus disease 2019 in metropolitan Detroit, a high proportion were admitted and required intensive care unit admission and invasive mechanical ventilation with a mortality rate of 40.4% among patients in the intensive care unit.

## Teaser

This case series describes the clinical characteristics and outcomes of patients with coronavirus disease 2019 (COVID-19) at a health system in metropolitan Detroit, Michigan, and provides a comparative analysis of hospitalized and ambulatory patient populations.

## Introduction

An outbreak caused by a novel coronavirus, severe acute respiratory syndrome coronavirus 2 (SARS-CoV-2), was reported in Wuhan, China in late December 2019.^1^ Three months later, coronavirus disease 2019 (COVID-19), the disease caused by SARS-CoV-2, was declared a pandemic by World Health Organization, and as of May 14, 2020, more than 4.3 million cases of COVID-19 and 297 000 deaths were reported.^2^ Since it was first detected in Washington State on January 20, 2020, the US now leads the world with more than 1.39 million confirmed COVID-19 cases and 84 000 deaths. All 50 states have reported cases of COVID-19, with most states reporting community spread.^3^

Early reports have suggested an incubation period of 2 to 14 days, with clinical presentations ranging from mild infection to severe disease to fatal illness.^3,4,5^ The most commonly reported symptoms are cough, fever, and dyspnea.^6,7,8,9^ Myalgia and gastrointestinal symptoms, including diarrhea and nausea or vomiting, are also common.^8^ Recent reports^6,9,10,11^ suggest that approximately 14% to 29% of hospitalized patients with COVID-19 pneumonia require intensive care, primarily for respiratory support in the setting of hypoxic respiratory failure, with acute respiratory distress syndrome (ARDS) developing in 33% of hospitalized patients at a median time from symptom onset of 8 days.^9^ In these reports,^6,8^ critically ill patients were older, more likely to be male, and to have underlying comorbidities. The mortality rate ranged from 8.7% to 21% among those patients admitted with pneumonia.^6,9,10,11^

On March 10, 2020, the state of Michigan confirmed its first 2 cases of COVID-19, and in the days since, the case number has increased to 49 582 with 4787 (10%) deaths, amounting to the highest case fatality rate in the nation.^3^ Metropolitan Detroit, which includes Oakland, Macomb, and Wayne counties, was at the epicenter of the outbreak, accounting for 80% of cases and 86% of deaths statewide. The city of Detroit accounted for almost one-third of all confirmed cases and deaths.^12^ The objective of this study is to describe the clinical characteristics and outcomes of the first 463 patients with COVID-19 evaluated at the Henry Ford Health System (HFHS), a 5-hospital system that serves metropolitan Detroit, and to perform a comparative analysis of hospitalized and ambulatory patient populations.

## Methods

### Study Design and Participants

 The HFHS institutional review board approved patient data review with waiver of consent because the data were deidentified. This is a case series that includes consecutive adult patients evaluated in HFHS from March 9, 2020 (date of first specimen tested), to March 27, 2020, and diagnosed with COVID-19. The HFHS is a comprehensive, integrated, health care organization that includes 5 hospitals and 9 emergency departments (EDs) in Southeast Michigan. Its 877-bed flagship hospital, Henry Ford Hospital, serves a large urban, mostly African American population in metropolitan Detroit. Patients were triaged on the basis of clinician judgment. All patients included in the study had SARS-CoV-2 infection confirmed by positive polymerase chain reaction testing of nasopharyngeal specimens. All clinical outcomes were monitored for 30 days.

### Data Collection and Definitions

A trained team of physicians and research associates performed a retrospective review of the electronic health records to obtain data on a standardized data collection form. Demographic data, underlying comorbidities, symptoms and signs at presentation, complications, treatment, and outcomes were collected and evaluated. Race/ethnicity data were collected in electronic health records by self-report using standard classification.^13^ Severe obesity (defined as body mass index [BMI] ≥40; BMI is calculated as weight in kilograms divided by height in meters squared) and ARDS were defined in accordance with the definition established by National Institute of Health^14^ and Berlin Definition,^15^ respectively. Acute kidney injury was diagnosed according to the Kidney Disease: Improving Global Outcomes definition.^16^ Cardiac injury was characterized by the presence of serum levels of high-sensitivity cardiac troponin I above the 99th percentile upper reference limit.

Complications and treatment included acute kidney injury requiring renal replacement therapy, hypoxic respiratory failure and/or ARDS requiring invasive mechanical ventilation (IMV), and shock requiring vasopressor treatment. Outcomes included discharge from the ED, length of stay, discharge disposition, and 30-day readmission and mortality rates.

### Laboratory and Radiographic Studies

Methods for laboratory confirmation of SARS-CoV-2 infection have been described elsewhere.^17^ Influenza polymerase chain reaction testing was also performed for all patients. Routine blood examinations included daily complete blood count with differential, basic metabolic panel, liver function tests, ferritin, magnesium, C-reactive protein, lactate dehydrogenase, and creatine phosphokinase, in addition to high-sensitivity cardiac troponin I and D-dimer every 48 hours. Furthermore, routine laboratory evaluation included interleukin-6 and disseminated intravascular coagulation panel when available for patients admitted to the intensive care unit (ICU). A chest radiograph and subsequent radiographs were obtained at baseline and as determined clinically by health care practitioners on case-by-case basis.

### Statistical Analysis

Continuous variables were described as median (interquartile range [IQR]) or mean (SD), and categorical variables were described as frequency rates and percentages. The *U* test or *t* test was used for continuous variables, whereas the χ^2^ or Fisher exact test was used for categorical variables. Comparisons between cohorts were analyzed using univariable analysis. Multivariable logistic regression was performed to model the association of demographic characteristics and comorbidities with ventilator-dependent respiratory failure. Multivariable regression was also performed to model the association of demographic characteristics and comorbidities with the need for ICU care at any time during the hospitalization. Race (African American or non–African American) was included in these models because of the high proportion of African American patients in Detroit. Demographic characteristics or comorbidities in which univariable analysis yielded *P* < .10 were included. Finally, multivariable logistic regression analysis of the associations of age older than 60 years, African American race, and sex with mortality was performed. At the time of analysis, mortality data were complete to 30 days after the initial encounter. We did not perform a formal sample size calculation for the study because the primary objective was to describe the characteristics of the initial surge of patients positive for SARS-CoV-2 within our health system. A 2-sided α < .05 was considered statistically significant. Odds ratios (ORs) with 95% CIs were reported for all models. Statistical analyses were performed using SAS statistical software version 9.4 (SAS Institute). Data analysis was performed from March to April 2020.

## Results

During the study period, 477 of 1459 patients (32.7%) tested positive for SARS-CoV-2 at HFHS. The first 2 reported cases at HFHS were on March 11, 2020. Of the 477 patients, 14 were excluded from the study because of a lack of demographic and baseline data. Most patients were African American (334 patients [72.1%]) and female (259 patients [55.9%]) with mean (SD) age of 57.5 (16.8) years. Most patients (435 [94.0%]) had at least 1 comorbidity, including hypertension (295 patients [63.7%]), chronic kidney disease (182 patients [39.3%]), and diabetes (178 patients [38.4%]). The mean (SD) number of comorbidities of admitted patients was 3.2 (1.8) compared with 1.9 (1.7) in patients discharged from the ED (difference, 1.3; 95% CI, 0.96-1.72; *P* < .001). Eighty-one admitted patients (22.8%) had prior ED visits for their symptoms compared with none of those who did not require hospitalization; more than one-fourth of all patients (124 patients [26.8%]) had known exposure to someone with COVID-19. Of note, there were no coinfections in patients who were not admitted; among hospitalized patients, 1 case of respiratory syncytial virus in the general practice unit (GPU) and 4 cases of influenza (2 in the GPU and 2 in the ICU [<1% of coinfections]) were detected. Table 1 summarizes the baseline clinical characteristics of the overall cohort.

**Table 1.** Baseline Characteristics of Patients With Coronavirus Disease 2019 at Presentation

Characteristic	Patients, No. (%)			*P* value
	All (N = 463)	Discharged home (n = 108)	Hospital admission (n = 355)	
Demographic characteristics				
Age, mean (SD), y	57.5 (16.8)	44.8 (15.1)	61.4 (15.4)	.005
Age >60 y	218 (47.1)	16 (14.8)	202 (56.9)	<.001
African American race	334 (72.1)	74 (68.5)	260 (73.2)	.34
Female	259 (55.9)	69 (63.9)	190 (53.5)	.06
Known exposure	124 (26.8)	31 (28.7)	93 (26.2)	.61
Prior emergency department visit in past 7 d	81 (17.5)	0	81 (22.8)	<.001
Symptoms				
Cough	347 (74.9)	87 (80.6)	260 (73.2)	.14
Nasal congestion	113 (24.5)	38 (35.2)	75 (21.2)	.003
Dyspnea	282 (60.9)	40 (37.0)	242 (68.2)	<.001
Fever	315 (68.0)	75 (69.4)	240 (67.6)	.72
Headache	74 (16.0)	28 (25.9)	46 (13.0)	.001
Myalgias	194 (42.0)	46 (42.6)	148 (41.8)	.89
Anorexia	100 (21.7)	10 (9.3)	90 (25.4)	<.001
Nausea	94 (20.4)	12 (11.1)	82 (23.2)	.007
Vomiting	53 (11.5)	8 (7.4)	45 (12.7)	.13
Diarrhea	100 (21.7)	14 (13.1)	86 (24.3)	.01
Comorbidities				
Asthma	73 (15.8)	20 (18.5)	53 (14.9)	.37
Chronic obstructive pulmonary disease	49 (10.6)	8 (7.4)	41 (11.6)	.22
Obstructive sleep apnea	57 (12.3)	8 (7.4)	49 (13.8)	.08
Diabetes	178 (38.4)	22 (20.4)	156 (43.4)	<.001
Hypertension	295 (63.7)	37 (34.3)	258 (72.7)	<.001
Coronary artery disease	59 (12.7)	3 (2.7)	56 (15.8)	<.001
Congestive heart failure	49 (10.6)	3 (2.8)	46 (13.3)	.001
Chronic kidney disease	182 (39.3)	21 (19.4)	161 (45.4)	<.001
End-stage renal disease	26 (5.6)	2 (1.9)	24 (6.8)	.06
Cancer	49 (10.6)	6 (5.6)	43 (12.3)	.05
Rheumatologic disease	10 (2.2)	1 (0.9)	9 (2.5)	.47
Solid organ transplant	11 (2.4)	3 (2.8)	8 (2.3)	.72
Body mass index, mean (SD)^a^	33.0 (8.5)	31.0 (7.3)	33.6 (8.7)	.01
Any obesity	262 (57.6)	52 (48.2)	210 (59.2)	.04
Severe obesity	89 (19.2)	14 (13.0)	75 (21.3)	.06
Tobacco use	160 (34.8)	23 (21.9)	137 (38.6)	.002
Vital signs, median (interquartile range)				
Lowest emergency department oxygen saturation as measured by pulse oximetry, %	94 (90-96)	98 (96-99)	93 (88-95)	<.001
Heart rate, beats/min	96 (84-109)	96 (84-106)	96 (83-109)	.71
Temperature, °F	99.0 (98.0-100.0)	99.0 (98.0-99.5)	99.0 (98.0-100.0)	.97
Respiratory rate, breaths/min	19 (18-22)	18 (17-18)	20 (18-22)	.02
Baseline chest radiograph findings				
Unilateral infiltrate	62 (13.4)	7 (11.9)	55 (16.5)	<.001
Bilateral infiltrate	187 (40.3)	5 (8.5)	182 (54.7)	
Normal	105 (22.7)	41 (69.5)	63 (18.9)	
Baseline laboratory values, median (interquartile range)				
White blood cell count, cells/μL	5.8 (4.2-7.5)	6.1 (3.8-8.6)	5.8 (4.3-7.5)	.03
Absolute lymphocyte count, cells/μL	0.8 (0.6-1.2)	1.0 (0.7-1.6)	0.8 (0.6-1.1)	.03
Creatinine, mg/dL	1.1 (0.84-1.54)	0.85 (0.69-1.18)	1.12 (0.85-1.61)	.001
Aspartate aminotransferase, IU/L	30 (26-55)	26 (24-58)	35 (27-55)	<.001
High-sensitivity cardiac troponin I >99th percentile	107 (23.1)	2 (1.9)	105 (29.6)	<.001

SI conversion factors: To convert degrees Fahrenheit to degrees Celsius, subtract 32 and multiply by .5556; white blood cell count to ×10^9^/L, multiply by .001; lymphocytes to ×10^9^/L, multiply by .001; creatinine to micromoles per liter, multiply by 88.4; aspartate aminotransferase to microkatals per liter, multiply by 0.0167.

^a^ Body mass index is calculated as weight in kilograms divided by height in meters squared.

Among all patients, 355 (76.7%) required hospital admission (Figure). Common symptoms at presentation were cough (347 patients [74.9%]), fever (315 patients [68.0%]), and dyspnea (282 patients [60.9%]). Symptoms of dyspnea (242 patients [68.1%] vs 40 patients [37.0%]), anorexia (90 patients [25.4%] vs 10 patients [9.3%]), nausea (82 patients [23.1%] vs 12 patients [11.1%]), and diarrhea (86 patients [24.2%] vs 14 patients [12.9%]) were more common among admitted patients than among patients who were discharged home. Furthermore, age older than 60 years (202 patients [56.9%] vs 16 patients [14.8%]), severe obesity (75 patients [21.3%] vs 14 patients [12.9%]), diabetes (156 patients [43.9%] vs 22 patients [20.4%]), hypertension (258 patients [72.7%] vs 37 patients [34.3%]), coronary artery disease (56 patients [15.8%] vs 3 patients [2.7%]), chronic kidney disease (CKD) (161 patients [45.4%] vs 21 patients [19.4%]), and cancer (43 patients [12.1%] vs 6 patients [5.6%]) were more common among admitted patients than among patients who were discharged home. The mean (SD) BMI of hospitalized patients was 33.6 (8.7), with 26% of severely obese patients requiring ICU. Patients requiring hospital admission also had a higher incidence of bilateral infiltrates on chest radiographs (182 patients [54.7%] vs 5 patients [8.5%]), more pronounced lymphopenia (median lymphocyte count, 0.8 cells/μL [IQR, 0.6-1.1 cells/μL] vs 1.0 cells/μL [IQR, 0.7-1.6 cells/μL]; to convert to ×10^9^/L, multiply by .001), and elevated liver enzyme levels (median aspartate aminotransferase level, 35 IU/L [IQR, 27-55 IU/L] vs 26 IU/L [IQR, 24-58 IU/L]); to convert to microkatals per liter, multiply by 0.0167) (Table 1).

**Figure.**
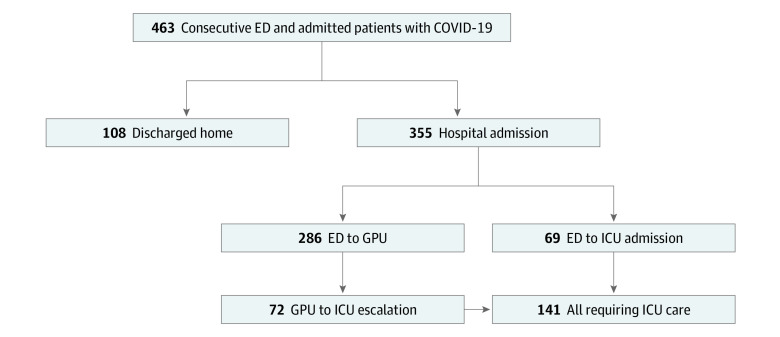
Disposition of Consecutive Patients With Confirmed Coronavirus Disease 2019 (COVID-19) ED indicates emergency department; GPU, general practice unit; and ICU, intensive care unit.

One hundred forty-one patients (39.7%) required ICU level care during their stay. On univariable analyses, those requiring ICU level care were more likely than patients in the GPU to be older than 60 years (92 patients [65.3%] vs 110 patients [51.4%]), male (80 patients [56.7%] vs 85 patients [39.7%]), and have comorbidities, including severe obesity (37 patients [26.2%] vs 38 patients [17.8%]), diabetes (73 patients [51.8%] vs 83 patients [38.8%]), hypertension (111 patients [78.7%] vs 147 patients [68.7%]), and CKD (83 patients [58.9%] vs 78 patients [36.5%]) (Table 2).

**Table 2.** Univariable Comparison of Patients With Coronavirus Disease 2019 Admitted to the Hospital, by Need for Care in the Intensive Care Unit

Characteristic	Patients, No. (%)		*P* value
	General practice unit (n = 214)	Intensive care unit (n = 141)	
Demographic characteristics			
Male	85 (39.7)	80 (56.7)	.002
African American race	156 (72.9)	104 (73.8)	.67
Age, mean (SD) y	59.8 (15.2)	63.8 (15.4)	.02
Age >60 y	110 (51.4)	92 (65.3)	.01
Prior emergency department visit	42 (19.6)	39 (27.7)	<.001
Symptoms			
Onset <7 d before admission	170 (79.4)	121 (85.8)	.65
Fever	155 (72.4)	95 (67.4)	.09
Dyspnea	140 (65.4)	111 (78.7)	.04
Headache	36 (16.7)	12 (8.5)	.02
Diarrhea	56 (26.2)	33 (23.4)	.43
Comorbid conditions			
Asthma	34 (15.9)	19 (13.5)	.53
Chronic obstructive pulmonary disease	23 (10.8)	18 (12.8)	.56
Obstructive sleep apnea	27 (12.6)	22 (15.6)	.43
Body mass index, mean (SD)^a^	33.0 (8.1)	34.4 (9.5)	.22
Any obesity	123 (57.6)	87 (61.7)	.43
Severe obesity	38 (17.8)	37 (26.2)	.06
Diabetes	83 (38.8)	73 (51.8)	.02
Hypertension	147 (68.7)	111 (78.7)	.04
Chronic kidney disease	78 (36.5)	83 (58.9)	<.001
End-stage renal disease	15 (7.0)	9 (6.4)	.99
Solid organ transplant	5 (2.3)	3 (2.1)	.99
Coronary artery disease	30 (14.0)	26 (18.4)	.26
Congestive heart failure	23 (10.8)	23 (16.6)	.11
Cancer	20 (9.4)	23 (16.6)	.04
Tobacco use	79 (36.9)	58 (41.1)	.42
Baseline respiratory vital signs			
Oxygen saturation as measured by pulse oximetry, median (interquartile range), %	94 (92-96)	89 (85-93)	<.001
Respiratory rate ≥22 breaths/min	47 (22.0)	47 (33.3)	.02
Bilateral infiltrate on chest radiograph	93 (43.5)	89 (63.1)	<.001
Baseline laboratory values, median (interquartile range)			
White blood cell, cells/μL	5.6 (4.1-7.2)	6.2 (4.5-7.8)	.05
Absolute lymphocyte count, cells/μL	0.9 (0.7-1.2)	0.8 (0.6-1.0)	.007
Creatinine, mg/dL	1.02 (0.82-1.40)	1.32 (0.98-1.92)	<.001
Aspartate aminotransferase, IU/L	31 (25-44)	44 (30-67)	<.001
Lactate dehydrogenase, IU/L	283 (235-375)	395 (296-526)	<.001
Ferritin, ng/mL	350 (171-718)	735 (347-1330)	<.001
D-dimer, μg/mL	0.95 (0.57-1.57)	1.43 (0.81-3.22)	<.001
Procalcitonin >0.5 ng/mL	20 (9.4)	31 (22.0)	<.001
High-sensitivity cardiac troponin I >99th percentile	47 (22.0)	58 (41.1)	<.001
Complications			
Acute respiratory distress syndrome	7 (3.3)	104 (73.8)	<.001
Acute kidney injury	61 (28.5)	98 (69.5)	<.001
Treatments			
Antibiotics	148 (69.2)	116 (82.3)	.01
Mechanical ventilation for respiratory failure	0	114 (80.8)	<.001
Vasopressors for shock	0	64 (45.4)	<.001
Renal replacement therapy	1 (0.5)	24 (17.0)	<.001
Length of stay, median (interquartile range), d	5 (3-7)	15 (9-23)	<.001
Outcomes			
Discharge disposition			
Home	183 (85.5)	49 (34.8)	<.001
In-house mortality	11 (5.1)	55 (39.0)	<.001
Placement in rehabilitation center	8 (3.7)	13 (9.2)	.66
Other	6 (2.8)	3 (2.1)	.75
30-d hospital readmission	27 (8.9)	2 (2.3)	<.001
30-d mortality	15 (7.0)	57 (40.4)	<.001

SI conversion factors: To convert white blood cell count to ×10^9^/L, multiply by .001; lymphocytes to ×10^9^/L, multiply by .001; creatinine to micromoles per liter, multiply by 88.4; aspartate aminotransferase to microkatals per liter, multiply by 0.0167; lactate dehydrogenase to microkatals per liter, multiply by 0.0167; ferritin to micrograms per liter, multiply by 1.0; D-dimer to nanomoles per liter, multiply by 5.476.

^a^ Body mass index is calculated as weight in kilograms divided by height in meters squared.

Signs and symptoms associated with the need for ICU care were dyspnea (111 patients in the ICU [78.7%] vs 140 patients in the GPU [65.4%]), tachypnea (47 patients in the ICU [33.3%] vs 47 patients in the GPU [22.0%]), and hypoxia (median oxygen saturation as measured by pulse oximetry, 89% [IQR, 85%-93%] for patients in the ICU vs 94% [IQR, 92%-96%] for patients in the GPU). On univariable analysis, symptoms of fever (155 patients [72.4%] vs 95 patients [67.4%]) and headache (36 patients [16.8%] vs 12 patients [8.5%]) at presentation were more common among patients in the GPU than patients in the ICU. Inflammatory marker and cardiac biomarkers were all higher in the ICU population than in the GPU population. Rates of complications were higher among patients in the ICU compared with patients in the GPU, including respiratory failure (114 patients [80.8%] vs 0 patients), acute kidney injury (98 patients [69.5%] vs 61 patients [28.5%]), ARDS (104 patients [73.8%] vs 7 patients [3.3%]), and shock (64 patients [45.4%] vs 0 patients). After adjusting for significant factors and race, male sex (OR, 2.0; 95% CI, 1.3-3.2; *P* = .001), severe obesity (OR, 2.0; 95% CI, 1.4-3.6; *P* = .02), and CKD (OR, 2.0; 95% CI, 1.3-3.3; *P* = .006) remained significantly associated with the need for ICU care (Table 3).

**Table 3.** Multivariable Analysis of Baseline Patient Characteristics Associated With Need for Intensive Care Unit Stay, Inclusive of All Admitted Patients

Characteristic	Odds ratio (95% CI)	*P* value
Male	2.0 (1.3-3.2)	.004
Age >60 y	1.6 (1.0-2.7)	.07
African American race	0.9 (0.5-1.6)	.78
Severe obesity	2.0 (1.4-3.6)	.02
Chronic kidney disease	2.0 (1.3-3.3)	.006
Cancer	1.9 (1.0-3.9)	.06
Diabetes	1.3 (0.8-2.2)	.25
Hypertension	1.0 (0.5-1.8)	.92
Coronary artery disease	1.1 (0.6-2.0)	.88

One-hundred four patients in the ICU (73.8%) developed ARDS and 114 (80.8%) required IMV for respiratory failure. The median time from symptom onset to need for IMV was 8 days (IQR, 6-10 days), and the median time to IMV after admission was 1 day (IQR, 0-3 days). Among the 286 patients initially admitted to the GPU, 72 (25.2%) were transferred to the ICU for escalation of care (Figure). The median time to ICU transfer from the time of admission was 2 days (IQR, 1-4 days). In a multivariable analysis, male sex (OR, 2.9; 95% CI, 1.7 to 4.8; *P* < .001), age older than 60 years (OR, 3.5; 95% CI, 1.9 to 6.4; *P* < .001), severe obesity (OR, 3.2; 95% CI, 1.7 to 6.0; *P* < .001), CKD (OR, 2.4; 95% CI, 1.4 to 4.2; *P* < .001), and cancer (OR, 2.5; 95% CI, 1.2 to 5.0; *P* = .01) were independently associated with the need for IMV (Table 4). There were 35 patients admitted younger than 40 years, 8 of whom required IMV. Of those requiring IMV, 5 (62.5%) had severe obesity compared with 7 (25.9%) of those who did not require IMV (difference, 36.6%; 95% CI, −5.5% to 67.2%; *P* = .09). Race was not associated with ICU admission or need for IMV.

**Table 4.** Multivariable Analysis of Baseline Patient Characteristics Associated With Need for Mechanical Ventilation, Inclusive of All Patients

Characteristic	Odds ratio (95% CI)	*P* value
Male	2.9 (1.7-4.8)	<.001
Age >60 y	3.5 (1.9-6.4)	<.001
African American race	0.7 (0.4-1.3)	.29
Severe obesity	3.2 (1.7-6.0)	<.001
Chronic kidney disease	2.4 (1.4-4.2)	.001
Cancer	2.5 (1.2-5.0)	.01
Diabetes	1.2 (0.7-2.0)	.58
Hypertension	0.9 (0.5-1.8)	.81
Coronary artery disease	1.3 (0.7-2.6)	.45
Congestive heart failure	0.7 (0.3-1.5)	.38
Tobacco use	1.1 (0.7-1.9)	.66

There was a difference in the length of stay between the 2 groups (median, 15 days [IQR, 9-23 days] for patients in the ICU vs 5 days [IQR, 3-7 days] for patients in the GPU). Overall, 262 (73.8%) hospitalized patients were discharged with median length of stay of 8.5 days (IQR, 4-11 days). In-house mortality was higher in the ICU (55 patients [39.0%]) than GPU (11 patients [5.1%]). Of the 11 patients in the GPU with in-hospital mortality, 2 died of pulseless electrical activity arrest, and 9 died of hypoxic respiratory failure because their code status was switched to “comfort care” at admission. (Table 2). More patients in the GPU (183 patients [85.5%]) than in the ICU (49 patients [34.8%]) were discharged to home (difference, 50.7%; 95% CI, 40.6%-59.6%; *P* < .001). Among patients initially discharged home from the ED, 4 (3.7%) were readmitted within 30 days and none died. Twenty-nine (11.2%) of those discharged from the hospital were readmitted within 30 days and 4 died. The overall 30-day mortality rate was 16.0% for the entire cohort and 20.0% for hospitalized patients; another patient died in the ED. Mortality in the ICU (57 patients [40.4%]) was significantly higher than in the GPU (15 patients [7.0%]) (difference, 33.4%; 95% CI, 24.3%-42.5%; *P* < .001); 52 patients requiring IMV (45.6%) died. Male sex (OR, 1.8; 95% CI, 1.1-3.1; *P* = .03) and age older than 60 years (OR, 5.3; 95% CI, 2.9-9.7; *P* < .001) were significantly associated with mortality, whereas African American race was not (OR, 0.98; 95% CI, 0.54-1.8; *P* = .86).

## Discussion

This study describes the demographic characteristics and clinical presentation of the initial 463 patients with COVID-19 evaluated in the HFHS, a multicenter academic institution in metropolitan Detroit. This study provides additional insight into the clinical presentation and outcomes of COVID-19 in an urban setting.

Compared with previous reports, most of our patients were African American and required hospitalization.^6,8,10^ However, our findings support the observations of earlier studies, which found a high percentage of hospitalized patients of advanced age with preexisting conditions, hypertension being the most common.^6,8,9,18^ COVID-19 rapidly spread throughout the state of Michigan and has disproportionately affected the African American population, who have high rates of comorbid conditions and mean BMI of 30.^19,20^ As of May 14, 2020, African American patients accounted for 32% of cases and 41% of deaths despite composing only 14% of the state’s population.^12,21^ This is in line with what has been described in other states.^22^ For example, in Louisiana, African American residents make up 32% of the state population, but accounted for more than 56% of COVID-19 deaths as of May 14, 2020.^23^

Factors such as lower wage positions and employment in critical infrastructure jobs, higher rates of poverty, lack of access to a personal vehicle and reliance on public transportation, and unstable or crowded housing conditions make preventive strategies such as social distancing and self-isolation or quarantine difficult to maintain.^24^ In Michigan, 27% of African American residents lived in poverty compared with 11% of white residents in 2019.^25^ These social determinants of health result in lack of health insurance and access to care, which may put patients at a disproportionately greater risk of acquiring infection and higher rates of complications from COVID-19.

Interestingly, more than one-half of cases and hospitalizations occurred among women, in contrast to previous reports.^6,8,9,18^ Women represent 51% and 53% of the Michigan and Detroit populations, respectively.^21,26^ In addition, they have a lower labor force participation rate (55%) and higher poverty rate (15% vs 12%) compared with their male counterparts (70%), according to the most recent data.^25^ Poverty can negatively affect family structure and increase health risks, all of which may have contributed to the higher infection rates in women.

The spectrum of disease described in our study is similar to those from the Centers for Disease Control and Prevention’s COVID-19–Associated Hospitalization Surveillance Network, the New York City area, and China.^5,7,9,27^ In the initial reports from Wuhan, China, during the early stages of the pandemic,^24^ shortness of breath was reported in 54% of patients and was associated with composite end point of admission to an ICU, use of mechanical ventilation, and death. A similar prevalence of dyspnea was reported in 21 critically ill patients in Washington State and in the COVID-19–Associated Hospitalization Surveillance Network database.^7,8^ In our series, dyspnea at presentation was associated with hospitalization and the need for ICU management. However, symptoms of fever and headache at presentation were more common among patients in the GPU than patients in the ICU on univariable analysis. A substantial proportion of patients presenting with gastrointestinal symptoms, including anorexia, nausea, and diarrhea, required hospitalization, similar to the data reported in the COVID-19–Associated Hospitalization Surveillance Network. Although we were testing for influenza, coinfection was very rare (<1%).

Compared with previous reports, a larger proportion of our hospitalized patients required ICU admission; 39.7% required ICU care compared with 14% in New York City area, 24% across the US, and up to 20% in reports from China.^6,9,11,24^ In the multivariable analysis, race was not associated with ICU admission or the need for IMV; however, male sex, severe obesity, and CKD were associated with need for ICU level care and IMV. A notable characteristic in our cohort was the prevalence of obesity; the mean BMI of hospitalized patients was 33.6, with 26% of severely obese patients requiring ICU. Severe obesity was independently associated with the need for mechanical ventilation (OR, 3.2; 95% CI, 1.7-6.0; *P* < .001). The association between obesity and need for IMV has been previously reported in patients with COVID-19.^25^ In a retrospective analysis^28^ of 124 patients in the ICU, almost one-half had BMI greater than 30, including 15% with BMI greater than or equal to 40. The mean BMI was 31 (range, 27.3-37.5) in patients requiring IMV compared with 27 (range, 25.3-30.8) in those who did not (*P* < .001).^28^ Moreover, in that study, BMI greater than 35 was independently associated with the need for IMV (OR, 7.36; 95% CI, 1.63-33.14; *P* = .02).^28^

The rate of complications, including acute kidney injury, hypoxic respiratory failure, and need for IMV, was higher and the length of stay was longer in our patient population than what was recently reported in New York City^6^; however, the rate of readmission was less than 10% in both cohorts. Overall mortality was in line with what has been reported in the US.^6,8,10^ Similarly, ICU mortality was higher than GPU mortality.^10^ Male sex and age older than 60 years were significantly associated with mortality but race was not.

### Limitations

This study has a few limitations. It was conducted a single large health system in Southeast Michigan. In addition, this case series has no control group, and the findings may not be generalizable to other populations.

## Conclusions

In this study of patients infected with SARS-CoV-2 in the metropolitan Detroit area, 76.7% of the patients who were infected were hospitalized and most of these patients were African American. The high prevalence of comorbidities and severe obesity in the population likely contributed to the disparities in morbidity associated with COVID-19.
